# Drivers of success in global health outcomes: A content analysis of Exemplar studies

**DOI:** 10.1371/journal.pgph.0003000

**Published:** 2024-05-09

**Authors:** Nadia Akseer, David E. Phillips, on behalf of the Exemplars in Global Health Partner Network

**Affiliations:** 1 Department of International Health, Johns Hopkins University, Baltimore, Maryland, United States of America; 2 Exemplars in Global Health, Gates Ventures, Seattle, Washington, United States of America; McGill University, CANADA

## Abstract

Applying a positive outlier lens is one effective approach for generating evidence to inform global health policy, program, and funding decisions. Exemplars in Global Health (EGH) is a program that studies positive outlier countries that have made extraordinary progress in health outcomes (despite limited resources) and disseminates their successes through multiple types of outputs. To date, EGH has studied, or is studying, 14 global health topics in 28 countries. This paper aims to identify findings, summarized as themes and sub-themes, that appear among all completed EGH studies. We developed a conceptual framework and used a content analysis approach to identify the top thematic areas that appear as drivers for programmatic success across EGH studies that were completed between June 2020-May 2023. The EGH studies (N = 31) spanned six topics including under-five child mortality (n = 6), childhood stunting (n = 5), community health workers (CHW) (n = 4), vaccine delivery (n = 3), COVID-19 response (n = 6), and newborn and maternal mortality reduction (n = 7) across 19 countries in sub-Saharan Africa, Latin America, South and Central Asia, and the Caribbean regions. Top drivers of success were defined as those critical or catalytic in achieving the intended outcome. Eight key drivers were identified: (1) efficient data collection and use for decision-making, (2) strong political commitment and health leadership, (3) effective stakeholder coordination, (4) a local, connected, and capacitated workforce, (5) intentional women’s empowerment and engagement, (6) effective adoption and implementation of national policies, (7) effective and sustainable financing, and (8) equitable, efficient outreach and targeting. These cross-cutting drivers span a broad range of development outcomes, sectors, and populations, and indicate a need to effectively integrate people, systems, and sectors to improve global health outcomes. Findings from this study aim to support peer learning among countries and support evidence-based decision-making for funders, policymakers, and other key stakeholders.

## Introduction

Global public health policy, program, and funding decisions ideally depend on timely and dependable evidence from a range of approaches, spanning experimental, quasi-experimental, and observational designs. Decision-makers in each of these sectors need a breadth of information about not only what interventions work, but also how they have been successfully delivered in different contexts.

One effective model for generating such evidence is known as a positive deviance, or positive outliers approach, whereby places, programs, or projects that have had exceptional success relative to their economic status are identified and studied for transferrable lessons [1]. This approach has been applied in global health in the past, with varying degrees of rigor [2–6].

**Fig 1:**
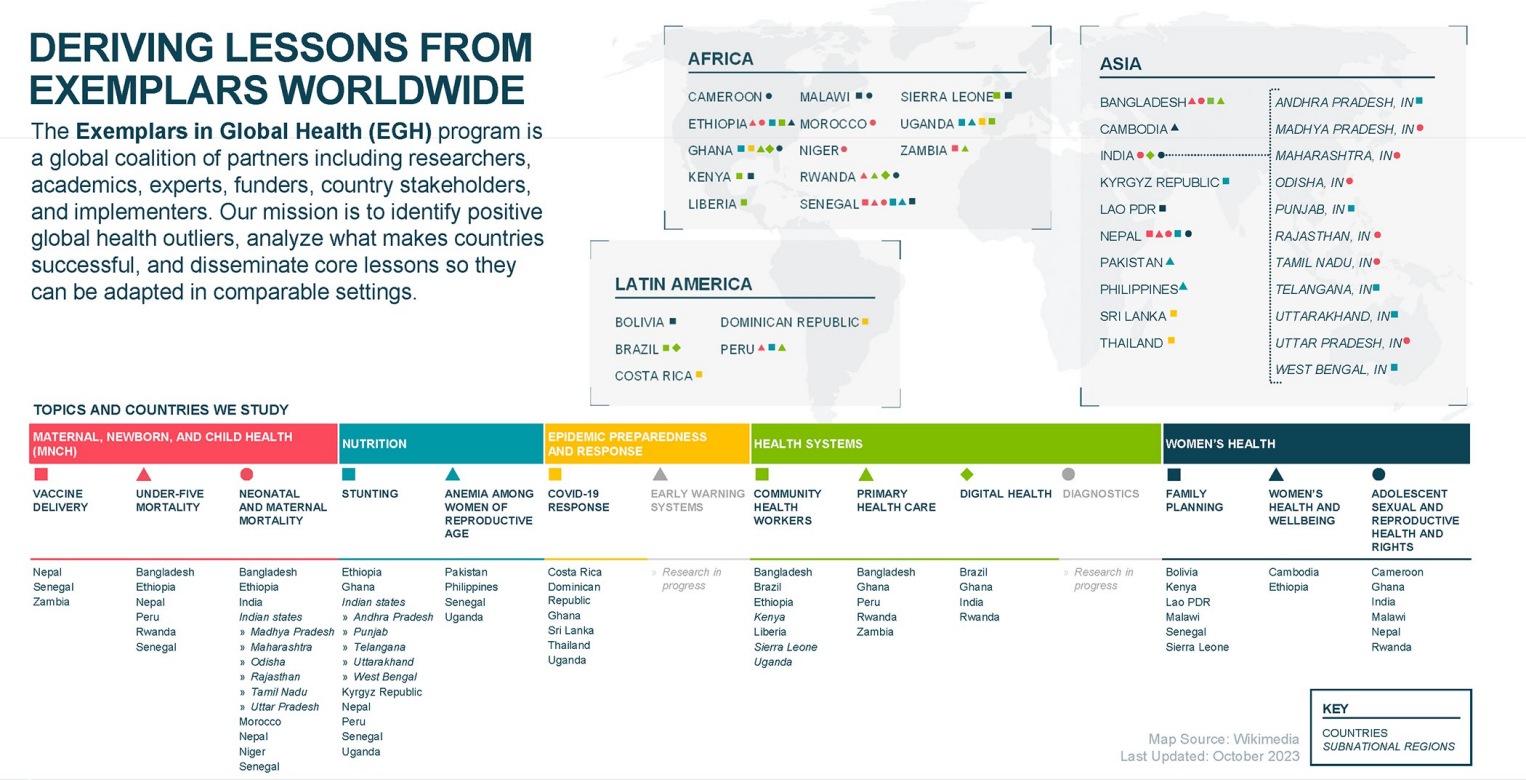
List of topics and countries studied by the Exemplars in global health program. Map Source: https://commons.m.wikimedia.org/wiki/File:Blank_world_map_Robinson_projection.svg.

In recent years, the Exemplars in Global Health (EGH) program has begun to identify and study positive outliers, with an aim of doing so as systematically and rigorously as possible [7]. EGH applies a consistent mixed methods design to study positive outlier countries and sub-national areas (referred to as Exemplars) via in-country research partners. To date, the EGH program has studied, or is currently studying, 14 major global health topics, which are determined through an inclusive, consultative process to identify information needs from country governments, norm-setting bodies, and donors. These topics include under-five mortality reduction, childhood stunting reduction [8–15], community health workers, vaccine delivery [16–26], neonatal and maternal mortality reduction [27, 28], COVID-19 response [29–36], primary health care, anemia among women of reproductive age, family planning, adolescent sexual and reproductive health and rights, women’s health and wellbeing, digital health, diagnostics, and early warning systems for diseases with epidemic or pandemic potential (Fig 1). Each topic’s consortium defines its own set of research questions about how its Exemplars achieved their success, conducts a study in each Exemplar country, and produces academic publications, case study reports, and narratives (content for the EGH web platform) from each study. Research questions are designed to be responsive to end-users through a participatory process of learning about information gaps among stakeholders and working with both topic and country experts to ensure the research addresses their needs. Research questions are answered through a combination of statistical analysis and qualitative research, guided by an *a priori*, evidenced-informed, conceptual framework. Topics are overseen by independent Technical Advisory Groups (TAGs), comprised of local and global thought leaders in the space, to ensure the quality, validity, and utility of the findings.

Since its inception in 2017, the EGH program has completed 31 studies (i.e., research on one of the topics listed above, in a specific country), spanning 19 countries under the first 6 of the 14 topics listed above (the remaining topics currently have research in progress), as well as research into subnational positive outliers in large countries such as India and Nigeria (Fig 1). Each EGH topic routinely produces a cross-country synthesis narrative that summarizes the overarching findings across countries studied for the same topic, but to date, no systematic effort has been taken to summarize findings across topics.

This paper aims to identify common themes and findings that resulted in unexpected health outcomes among the completed EGH studies. Specifically, we will present 1) the top thematic areas that appear across Exemplar studies (i.e., highlighting “what” different Exemplars did to achieve impressive outcomes); and 2) the key sub-themes in each of the thematic areas (i.e., highlighting “how” the Exemplars achieved successes in each thematic area). In documenting these lessons, we aim to demonstrate cross-cutting drivers of exceptional performance in global health so that decision-makers throughout the landscape of global health organizations can adopt more effective practices.

## Materials and methods

### Study design and sampling

This qualitative study employed a descriptive content analysis approach to study key themes and patterns emerging from the EGH studies [37–47]. EGH studies used the positive deviance approach as described above; methods details have been published elsewhere [7] and in respective studies[9–14, 21, 22, 24–26, 28, 36] We used purposive sampling, considering all completed EGH studies (N = 31) from June 2020 to May 2023 for inclusion in this analysis. Studies were excluded if validation of final results was ongoing or if research partners requested to independently publish findings prior to this study. Studies covered the following topics: under-five child mortality (n = 6), childhood stunting (n = 5), community health workers (n = 4), vaccine delivery (n = 3), COVID-19 response (n = 6), and neonatal and maternal mortality reduction (n = 7) across 19 countries in sub-Saharan Africa, Central and South Asia, East Asia-Pacific, Latin America, and the Caribbean regions (Table 1).

**Table 1:** Exemplar countries included in content analysis.

Geography^a^	Case Studies	Population^b^ (2021)
**East Asia and Pacific**		
Thailand	Exemplars in Pandemic Response (EPR)	71.60M
**Europe and Central Asia**		
Kyrgyz Republic	Stunting	6.53M
**Latin America and the Caribbean**		
Brazil	Community Health Workers (CHW)	214.33M
Costa Rica	EPR	5.15M
Dominican Republic	EPR	11.12M
Peru	Under-Five Mortality (U5M), Stunting	33.72M
**Middle East and North Africa**		
Morocco	Neonatal and Maternal Mortality Reduction (NMR/MMR)	37.08M
**South Asia**		
Bangladesh	U5M, CHW, NMR/MMR	169.36M
India	NMR/MMR	1407.56M
Nepal	U5M, Stunting, Vaccine Delivery, NMR/MMR	30.03M
Sri Lanka	EPR	21.77M
**Sub-Saharan Africa**		
Ethiopia	U5M, Stunting, CHW, NMR/MMR	120.28M
Ghana	EPR	32.83M
Liberia	CHW	5.19M
Niger	NMR/MMR	25.25M
Rwanda	U5M	13.46M
Senegal	U5M, Stunting, Vaccine Delivery, NMR/MMR	16.88M
Uganda	EPR	45.85M
Zambia	Vaccine Delivery	19.47M

^a^Country regions are listed based on The World Bank classification.

^b^Population from United Nations Development Programme (UNDP).

### Data sources

We conducted a desk review of all completed EGH country case study reports, online platform narratives, and peer-reviewed publications for the 31 completed studies. This included a total of 70 documents across the six topics. In addition, we consulted EGH cross-country and in-country research partners that were engaged in the original studies (Table 2) as well as internal EGH research leads for additional contextual information.

**Table 2:** Exemplars in global health topic research partners.

Topic	Cross-Country Research Partner	Country	In-Country Research Partner
Community Health Workers (CHW)	Last Mile Health (LMH)	Bangladesh	BRAC, BRAC University and BRAC James P Grant School of Public Health
		Brazil	National School of Public Health–Oswaldo Cruz Foundation (FIOCRUZ)
		Ethiopia	International Institute of Primary Healthcare-Ethiopia (IIfPHC-E)
		Liberia	University of Liberia, School of Public Health
Exemplars in Pandemic Response (EPR)	Brown University School of Public Health; Johns Hopkins University	Costa Rica	INCAE Business School
		Dominican Republic	Fundacion Plentitud
		Sri Lanka	Institute for Health Policy
		Thailand	National Health Foundation
	Makerere University School of Public Health	Ghana	University of Ghana School of Public Health
		Uganda	Makerere University School of Public Health
Neonatal and Maternal Mortality Reduction (NMR/MMR)	Johns Hopkins University	Bangladesh	International Centre for Diarrhoeal Disease Research, Bangladesh (icddr,b)
		Niger	Niger National Institute of Statistics
	London School of Hygiene and Tropical Medicine	Morocco	Centre Sante Reproductrice
		Nepal	South Asian Institute for Policy Analysis
		Senegal	African Population and Health Research Center (APHRC)
	London School of Hygiene and Tropical Medicine; University of Manitoba	Ethiopia	Ethiopian Public Health Institute
	University of Manitoba	India	National Health Systems Resource Centre International Institute for Population Sciences (NHSRC, IIPS); India Health Action Trust (IHAT)
Stunting	The Hospital for Sick Children (SickKids)	Ethiopia	Addis Ababa University (AAU)
		Kyrgyz Republic	University of Central Asia (UCA)
		Nepal	Nepal Public Health Foundation (NPHF)
		Peru	Universidad Peruana Cayetano Heredia (UPCH)
		Senegal	Université Cheikh Anta Diop (University of Dakar)
Under-Five Mortality (U5M)	University of Global Health Equity (UGHE); Northwestern University	Bangladesh	International Centre for Diarrhoeal Disease Research, Bangladesh (icddr,b)
		Ethiopia	Monitoring, Evaluation, Research and Quality Improvement Consultancy (MERQ)
		Nepal	Nepal Public Health Foundation
		Peru	Universidad Peruana Cayetano Heredia (UPCH)
		Rwanda	University of Global Health Equity (UGHE)
		Senegal	National Agency of Statistics and Demography of Senegal (ANSD)
Vaccine Delivery (VxDel)	Emory University	Nepal	Center for Molecular Dynamics Nepal (CMDN)
		Senegal	Institute for Health Research, Epidemiological Surveillance and Training (IRESSEF)
		Zambia	Center for Family Health Research in Zambia (CFHRZ)

### Conceptual framework

**Fig 2:**
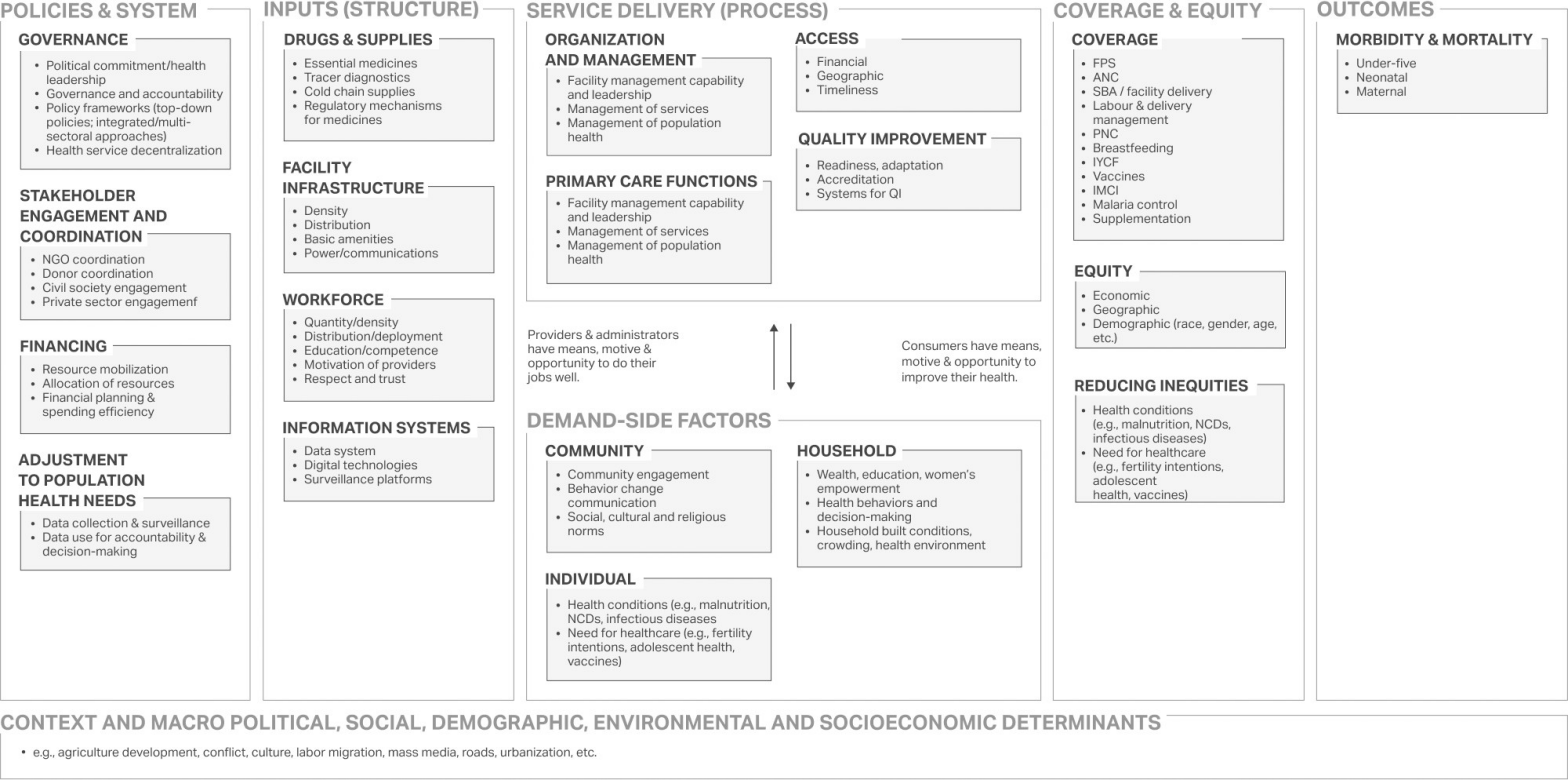
EGH cross-topic synthesis analysis framework.

We developed a conceptual framework that adapts several existing health systems frameworks [48, 49] as well as frameworks used for the included EGH topics (Fig 2). These frameworks were selected as they were designed or adapted by EGH research experts for the respective topics that were included in this study. All unique domains and sub-domains across these frameworks were included in the overall framework, and any duplicated concepts were harmonized (e.g., terminology, constructs reduced or expanded) as needed. The framework was developed *a priori* to guide the analysis for this study and was iterated with EGH research teams and research partners to refine the final set of concepts, nomenclature, and definitions. The framework identifies key factors of successful intervention delivery and illustrates the relationship between distal policies and systems, health system inputs (e.g., health facility resources and infrastructure), service delivery processes and coverage, and equity on key health outcomes. Underlying each of these spheres of influence is the macro political, social, environmental, and economic context of a country.

### Analysis

We undertook a conceptual content analysis approach, which maps and quantifies the presence of words, themes, or concepts across data, to identify any emerging patterns or themes [50–52]. Content analysis is a flexible approach that is well-suited for analyzing data on multifaceted phenomena and is increasingly used in nursing [53–55], public health [37, 38, 56, 57], psychiatry [39–42], and health informatics [43, 44], among other areas [45, 46]. We initially utilized an inductive approach (i.e., open coding), followed by deductive coding that was guided by the conceptual framework [47]. After coding, data were then categorized and mapped in larger categories and grouped into emergent themes to identify top drivers of the successes achieved in Exemplar studies (i.e., factors that were critical or catalytic in achieving the intended outcome) [47].

Emergent themes were quantified (Yes/No– 0/1) and arranged in a master database aligned with the conceptual framework (S2 File). An indicator was developed to measure the presence (%) of the theme across Exemplar studies as follows: number of studies where the theme appeared in publications, case study reports, and/or narratives as a top driver of the successes / total number of Exemplar studies. A threshold (>75%) was set to select top drivers across studies. This threshold was set by examining the distribution of percentages among drivers and identifying a rough threshold above which only a small number of drivers remained. In this quantification, each Exemplar study was given equal weight to avoid inaccurately overrepresenting findings from some countries, i.e., if countries were weighted by population, findings from case studies in India would significantly outweigh those from other countries with lower populations. Some themes were not considered for all studies, especially if the theme was not studied (e.g., women’s education and empowerment in COVID-19 response) or if the theme was part of the outcome (e.g., existence of a CHW program could not be a top driver in studies focused on mapping drivers of the CHW program’s success). The analysis process was led by NA with continuous consultation with internal EGH research teams and additional consultation with research teams responsible for the original studies. Any discrepancies were discussed and resolved by cross-referencing study documents and consultations with research partners, where needed. Data coding was performed manually, and descriptive analyses were executed in Excel 2023 and Stata version 15.

The checklist for Standards for Reporting Qualitative Research (SRQR) was also consulted (S3 File).

Sensitivity analyses were also conducted using the country as the unit of analysis to account for countries that had multiple studies (e.g., Senegal was a region of study in stunting, under-five child mortality, vaccine delivery, and neonatal and maternal mortality reduction), which were given one weight in the final analyses to ensure each country was equally represented (S2 File). A theme was listed as a top driver if it emerged in any one of the included topics for the country.

### Validation

A technical advisory group (TAG) comprised of experts, including research partners and principal investigators from the selected studies, were assembled to support validation of this EGH cross-topic synthesis. The TAG had research and policy expertise in key domains as relevant to this study including child nutrition, maternal, newborn and child mortality, community health workers, vaccine delivery systems and pandemic response/COVID-19. The TAG was engaged from the outset of the study. They joined a TAG meeting where the study approach and goals were shared. The TAG subsequently reviewed the content analysis methodology, development of broader framework, the emergent themes, and the final results in an iterative way over email between 2021 and 2023. Feedback from the TAG was discussed and reflected into the work as appropriate. Any discrepant suggestions or feedback was resolved by open dialogue between study researchers and TAG members until agreement was reached.

## Results

Countries included in the content analysis represented Sub-Saharan Africa (n = 8), South Asia (n = 4), Latin America and the Caribbean (n = 4), Middle East and North Africa (n = 1), Europe and Central Asia (n = 1), and East Asia and Pacific (n = 1) and were largely lower middle income (n = 14) and upper middle income countries (n = 5) in 2023 (Table 1). The median population size across countries was approximately 30 million, ranging from 5 million (Costa Rica) to nearly 1.4 billion (India).

**Fig 3:**
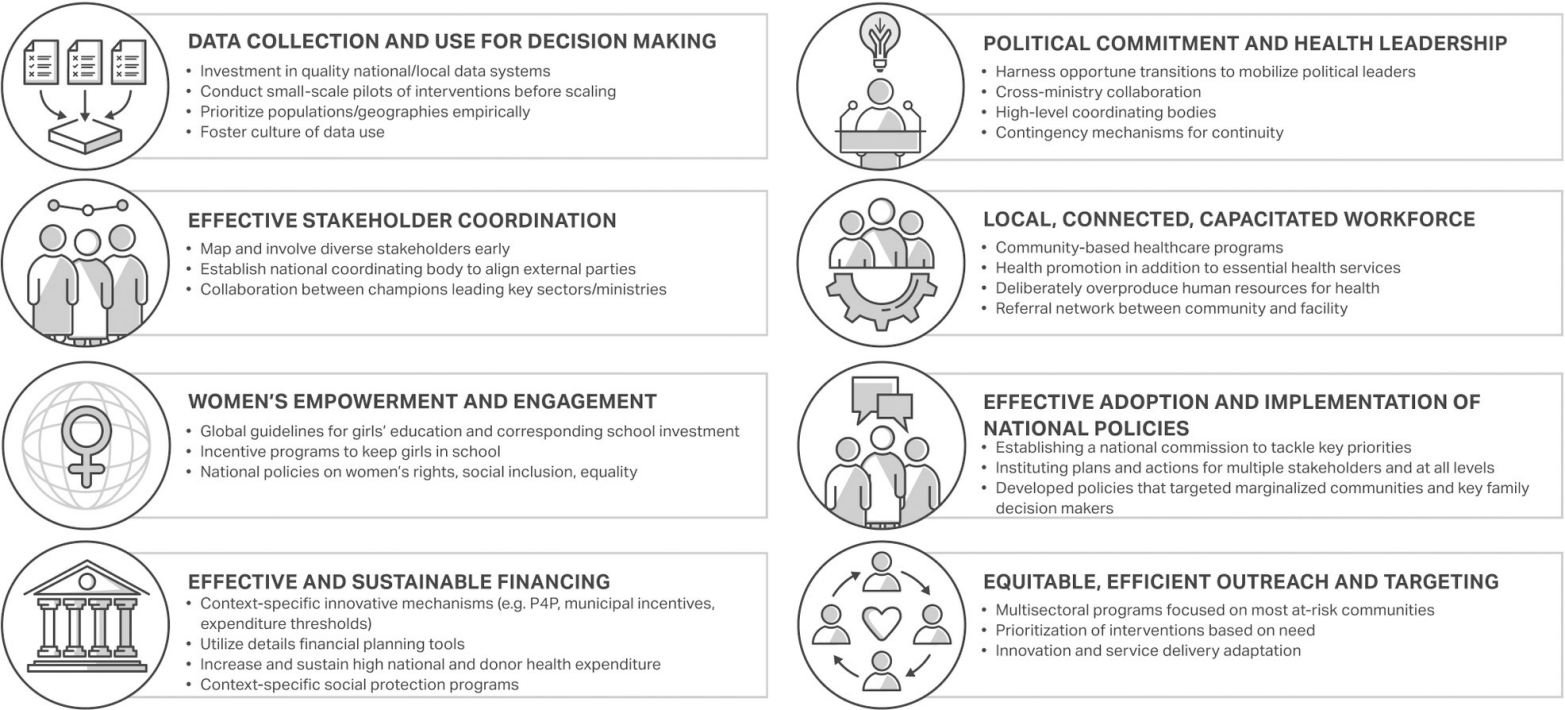
Key themes and sub-themes from EGH studies.

A total of eight themes emerged, as presented in Fig 3. Sensitivity analyses based on country identified most of the same themes.

Key drivers of success included: (1) efficient data collection and use for decision-making, (2) strong political commitment and health leadership, (3) effective stakeholder coordination, (4) a local, connected, and capacitated workforce, (5) intentional women’s empowerment and engagement, (6) effective adoption and implementation of national policies, (7) effective and sustainable financing, and (8) equitable, efficient outreach and targeting.

### Efficient data collection and use for decision-making

In 28/31 (90%) studies, efficient data collection was used to make strategic decisions. Exemplar countries *invested in high-quality national and local data collection and analysis systems*. An example of this was observed in Ethiopia, which prioritized their system’s interoperability by investing in national data collection structures such as the Health Management Information System (HMIS) (rolled out in 2008), an electronic version (mid-2010s), the establishment of the Central Statistical Agency (2005), and the National Statistical Development Strategies (2009, 2015) [58]. Other settings *conducted small-scale or local research studies to pilot interventions before scale-up*, complementing use of routine data. For example, researchers and health professionals relied heavily on local research to validate global best practices before considering scale-up of interventions in Nepal; the success in locally designed and implemented research informed adoption of chlorhexidine as a cord care treatment and the vitamin A campaign [59]. Additionally, *using data to identify priorities and tailor interventions to local contexts* was a key driver in many studies, including Zambia’s adapted “reaching every district” (RED) strategy, which strengthened community-level immunization campaigns using an inclusive, facility-level microplanning that included local leaders to successfully reach remote communities [22, 60]. Lastly, *fostering a culture of data for decision making* proved critical, for example, in Ghana during COVID-19 where the country relied on existing (i.e., District Health Information Management System, DHIMS) and newly-developed systems (i.e., The Surveillance Outbreak and Response Management and Analysis System) for rapid data sharing with key health officials, health workers, and the public, as well as real-time tracking of new cases, allowing key decision makers to swiftly adjust mitigation strategies [61]. Despite these success factors, several countries also noted that challenges and opportunities remained to improve data quality, including in improving completeness and representation.

### Strong political commitment and health leadership

In 28/31 (90%) of Exemplar studies, strong political commitment and government leadership was critical for achieving outcomes. This was often measured through the formation of new entities or national plans. A key strength of successful programs was the ability to *seize windows of opportunity to mobilize leaders* to advocate for change. For example, when Dominican Republic’s new president and administration were taking office in August 2020, they mobilized the country towards a common goal of protecting the population from COVID-19 through mass vaccination while gradually reopening the economy [62]. Other programs *developed high-level coordinating bodies* for key strategies and plans such as Senegal’s *Cellule de Lutte Contre la Malnutrition* (Coordination Unit for the Fight Against Malnutrition, CLM) [63]. Located in and chaired by the prime minister’s office, rather than within the Ministry of Health, brought together representatives of key stakeholders, the Ministry of Health, and additional ministries of Education, Agriculture, and Industry, all of which are involved in nutrition. This elevated its prominence, allowing for the successful launch of the multisectoral *Programme de Renforcement de Nutrition* (Nutrition Enhancement Program) in 2002, which impressively established 5,000 community-based nutrition workers delivered nationally. Another factor of success was the *institution of contingency plans and processes to ensure continuity in service delivery during crises* as was critical in Liberia when in the aftermath of the Ebola epidemic, the government, partners, and donors moved swiftly to provide basic health care to all citizens and establish an early warning system to mitigate any potential future epidemics [64].

### Effective stakeholder coordination

In 26/31 (84%) of studies, effective stakeholder coordination was identified as a top driver of success. A key strategy was engaging partners meaningfully throughout the programs. In Bangladesh, a CHW program intentionally engaged key implementing partners such as the International Centre for Diarrheal Disease Research, Bangladesh (icddr,b), BRAC, and CARE to engage in the design, funding, implementation, and evaluation of various interventions, including family planning, pediatric diarrhea, and CHW capacity-building [65]. Another success factor was the institutionalization of stakeholder coordination in the form of official coordinating bodies. In Senegal, the Ministry of Health and Social Action’s Reproductive, Maternal, Newborn, and Child Health cluster–which included the United Nations Children’s Fund (UNICEF), World Health Organization (WHO), and the World Bank–led to an efficient and impactful strategy that helped drive the significant reduction in under-five child mortality [66]. Others engaged program champions to ensure stakeholder accountability and durability was key, like in Peru’s progress against stunting where regional leaders publicly pledged for stunting reduction through financial incentive programs such as the *Fondo de Estimulo al Desempeño y Logro de Resultados Sociales* (Fund to Stimulate Performance and Achievement of Social Results, RED) program [67].

### Local, connected, and capacitated workforce

In 26/31 (84%) of Exemplar studies, having a strong, local, connected, and well-equipped workforce was key to achieving outcomes. This workforce was often a mix of paid and unpaid health professionals. Firstly, investments in *strong community-based healthcare* were pivotal. This sub-theme emerged in a majority of the case studies reviewed. Nepal’s Female Community Health Volunteer (FCHV) program was designed to extend health services to the most remote and hard-to-reach communities and very quickly became a central pillar of the health system, providing a source for local knowledge in addition to a critical link between last mile communities and the public health system [68]. During the COVID-19 pandemic, Thailand preemptively appointed nearly 40,000 contract nurses, frontline health workers, and other short-term health workers and deployed them into communities; this initiative was successful in preventing health worker shortages and ensuring health professionals could be available and designated for the COVID-19 wards as needed [69]. Exemplar countries were also successful at *expanding the role of health workers to include service provision and health promotion* when needed. Senegal’s success in creating an unusually diverse community health system that meets a wide range of community needs is one example. Innovations included health huts, *agents de santé communautaires* (community health workers, providing a wide range of preventative and curative services and referring complicated cases to higher-level facilities), *matrones* (matrons, focused on maternal and newborn health), *dispensateurs de santé à domicile* (home health providers, supporting curative services in the home for conditions such as malaria, diarrheal diseases, and acute respiratory infections), *relais communautaires* (community relays, covering a wide range of issues in the community), and *bajenu gox* (“godmothers”) [66]. *Creating educational programs to train sufficient cadres of doctors*, *nurses*, *and other health workers* was also a key driver in Exemplar countries. To address the problem of many Ethiopian doctors leaving the country to practice abroad where salaries are higher, the Ministry of Health adopted a policy of “deliberately overproducing” healthcare professionals [70]. In 2012, 13 new medical schools opened, more than doubling the country’s total. As of 2016, there were 33 medical schools, and those schools graduated approximately 3,000 new doctors. Exemplar countries also had success in s*trengthening referral networks between community and facility-based health care* and building integrated teams. A pillar in Brazil’s primary health care system is the Family Health Team (consisting of a nurse, nurse assistant, physician, and four to six CHWs). Brazil embedded CHWs within the Family Health Team to strengthen CHWs’ abilities to link communities to a continuum of preventive and curative care provided by the Family Health Team at nearby primary health care facilities and to leverage the deep knowledge of CHWs as community insiders to better serve families [71].

### Women’s empowerment and engagement

In 20/25 (80%) of studies, the education and empowerment of girls and women were key facilitators in the successes achieved. As a note, the six COVID-19 response studies did not study this driver and hence were excluded for this theme. Many Exemplar countries were *investing and implementing best practices in girls’ education*. Senegal launched a flurry of education policies and investments, including the National Action Plan for Education for All and the Ten-Year Education and Training Plan, both of which were structured according to the Dakar Framework (2000–2015) and prioritized both girls and boys in these initiatives [63]. Given high drop-out rates among adolescent girls, some Exemplars *created incentive programs to keep girls in school*. Bangladesh’s Female Secondary School Stipend Project was successful in improving girls’ secondary school enrollment and retention [72]. It provided girls in rural areas with stipends for attending school at least 75 percent of the time, maintaining passing grades, and delaying marriage until the completion of secondary school or their 18^th^ birthday. *Adopting policies and devising strategies on women’s rights*, *social inclusion*, *and equality* were also key drivers in Exemplar countries. Nepal led in this area by establishing a Ministry of Women and Social Welfare (1990s), a National Plan of Action for Gender Equality and Women’s Empowerment (1997), and the Health Sector Gender Equality and Social Inclusion Strategy (2010) [59]–each of which played a key role in improving women’s representation in society and public life, including in parliament in Nepal.

### Effective adoption and implementation of national policies

Another top driver (24/31, 77% of studies) was the effectiveness and scale at which key national or subnational laws, legislations, policies, or large-scale programs were adopted at a country level and successfully scaled to improve outcomes. This was often accomplished via bidirectionality of top-down and bottom-up approaches. One key strategy was to *establish a national commission to tackle key priorities*. Supported with a national champion, Morocco was able to establish a national commission to prioritize efforts to reduce maternal mortality; the commission developed the strategy to support the plan *d’accélération de la réduction de la mortalité maternelle* (accelerating the reduction of maternal mortality) (2008 to 2012), which was critical in improving maternal survival [73]. Another key approach was to *institute effective plans and actions for stakeholders at all levels*. In response to the COVID-19 pandemic, Sri Lanka coordinated a top-down response where national centers, such as the National Operations Centre for Prevention of COVID-19 Outbreak and task forces (i.e., Presidential Task Force for the COVID-19 response), were established to focus on preventive and management measures to ensure that health care and other services are well-geared to serve the general public [74]. To combat COVID-19, the Ugandan Ministry of Health also activated a centralized Public Health Emergency Operations Center (PHEOC) and National Task Force comprising public health experts and representatives from government agencies [75]. These multisectoral and multilevel coordination committees offered clear technical guidance and direction to district and local officials and health care providers and helped translate emerging data into national policy and practice. Several Exemplars also developed policies at the highest level that *focused on priorities for marginalized communities or key family decision-makers*, such as Niger’s *écoles des maris*, loosely translated as “husband schools” [73]. These schools focus on educating men in the household on critical maternal and child health and nutrition issues to improve healthcare decision-making for their families.

### Effective and sustainable financing

In 25/31 (81%) of Exemplar studies, effective financing was key; countries found ways to extend use of their funds in many ways, including aligning funders to reduce duplication, using sophisticated financial planning tools to model spending scenarios, and implementing financing models that incentivize performance. Brazil’s c*reation of innovative financing mechanisms* expedited implementation of the Family Health Strategy by ensuring a steady revenue stream for participating municipalities through three innovative funding mechanisms: thresholds for health care spending, financial incentives for municipalities to adopt the Family Health Strategy and benefit the poorest municipalities, and a pay-for-performance program to improve quality [76]. In Liberia, *using detailed financial planning tools* was essential. During the early planning phase of Liberia’s community health assistant program, the government developed detailed financial costing and sustainability modeling to track and study three key variables: potential costs, potential benefits, and potential funding [77]. With these tools, those working in Liberia could understand, at an extremely granular level, the costs and benefits of various elements of the program, as well as who might be willing to pay for them, and for how long. The government of Rwanda consistently maintained relatively *high levels of national health expenditure* by keeping health a priority and including the ambitious rollout of a national health insurance plan [78]. Meanwhile, Rwanda successfully encouraged and coordinated foreign donors and partners to follow—and contribute to—a nationally-led agenda. This has enabled Rwanda to retain national control of its own health policy while benefitting from foreign insight and technical assistance. Meanwhile, the Kyrgyz Republic started to reform its Soviet-era social protection system by re-designing *social protection programs based on local contexts*. Successful programs such as the Universal Monthly Benefit (UMB), a means-tested cash benefit for the poorest families with children between the ages of 18 months and 16 years was a result [79].

### Equitable, efficient outreach and targeting

In 24/31 (77%) of studies, addressing population health inequities through outreach and targeting was a key driver. *Implementing multisectoral programs focused on high-burden communities* was one effective approach. Peru’s *Crecer* (To Grow) program (launched in 2007) was a successful effort in consolidating the country’s existing poverty alleviation and nutrition programs. The program selected high-impact, evidence-based interventions and deployed them in key geographies (such as the poorest or hardest to reach) where they could make the most difference [67]. Several Exemplar countries also regularly used context-specific *innovations and adapted service delivery* to achieve optimal outcomes. To protect healthcare workers but also continue provision of essential healthcare services, Costa Rica, as part of its COVID-19 response, had 4,800 healthcare workers providing services remotely. Key services included online and telemedicine outpatient consultations and monitoring of asymptomatic COVID-19 patients by phone to reduce system burden [80]. Exemplar countries were also effective at *delivering interventions based on need*. In India, several states emphasized geographic targeting and developing tailored interventions based on need and evidence. District-level management infrastructure was leveraged to catalyze implementation of key maternal and newborn health programs tailored to local coverage and equity needs, while financial flexibility enabled effective and efficient resourcing [81].

## Discussion

Findings from this content analysis of Exemplar studies highlight cross-cutting drivers of success across broad health and development outcomes, including efficient data systems, effective leadership, effective stakeholder coordination, a capacitated workforce, women’s empowerment, a conducive national policy environment, sustainable financing, and outreach.

These findings generally align with previous efforts aimed at identifying drivers of global health improvements. The WHO’s Health Systems Framework identifies leadership and governance, including the creation of strategic policy frameworks, effective oversight, and coalition building, as health system building blocks [82]. Consistently, we have found that political commitment and health leadership as well as effective adoption and implementation of national policies are key drivers of health improvements. Also included in the WHO framework are a well-performing health workforce, functioning health information system, and adequate health funding that protects the public and provides effective goods and services [82]. This aligns with the identified themes of a local, connected, and capacitated workforce, data collection and use for decision-making, and effective and sustainable financing. The 2022 Sustainable Development Goals report also identified an urgent need for increased data investment as data collection costs rose and government funding for national statistics offices was cut during the pandemic [83]. The lessons learned from the Millennium Development Goals also reflected the findings that data are indispensable to the development agenda and that strong political commitment and more resources are needed to achieve targets [84].

The WHO’s Success Factors for Women’s and Children’s Health studies identified fast-track countries that were able to reduce maternal and child mortality, finding three effective approaches: engagement of multiple sectors, mobilization of partners across society using evidence for decision-making while maintaining accountability, and effective leadership through establishing guiding principles to orient and align stakeholder action [85, 86]. Our study corroborates these findings through the identified themes of political commitment through cross-ministry collaboration, effective stakeholder coordination, data collection for decision-making, and effective adoption and implementation of national policies with overarching and cross-cutting strategies. The Countdown to 2015 work on tracking maternal, newborn and child survival found that key drivers of increased coverage of life-saving interventions included financing, human resources, commodities, and favorable health policies [87]. Furthermore, Good Health at Low Cost, an initiative to identify why some countries outperform others in health and social outcomes, identified attributes of success related to good governance and political commitment with institutions that preserved knowledge gained and learned from experience to adapt despite limited resources [3]. Women’s empowerment, education, transport infrastructure, and resilience-building were also identified as components of success [3]. Finally, the WHO and UNICEF’s Primary Health Care (PHC) framework identifies four core strategic levers that include political commitment and leadership, governance and policy frameworks, funding and allocation of resources, and engagement of community and other stakeholders [48].

Notably, the findings pose some differences with the broader literature as well. Although other similar initiatives shared several themes with the findings reported here (e.g., political commitment, national policies, data collection, financing, stakeholder coordination, workforce, and women’s empowerment, as noted above), our study supports other drivers that are more nascent in the broader literature, such as equitable outreach and targeting. Community engagement, a strategic lever supported by the WHO and UNICEF PHC framework among other comparable efforts, was not listed among the primary eight drivers of this study. However, community engagement to define problems, solutions and priority actions did emerge as a common driver among EGH studies, albeit below the 75% threshold.

Our findings on the education and empowerment of girls and women being key to successes achieved are very timely and important. Indeed, some countries today, such as Afghanistan, grapple with issues around how to engage girls and women in their societies including in the education system, the workforce and in public life. This study of drivers of success across diverse outcomes and geographies around the world over a 20-year period underscore, yet again, the importance that girls and women’s education and empowerment has to countries achieving optimal health and survival outcomes. We hope that donors and national governments can consider these findings in country development strategies and action plans.

### Implications of findings

While the results of this study are not entirely new to the field, it does contribute meaningful information in at least two ways:

1. It reinforces a set of principles that ought to influence national health strategies by showing that they are present in top-performing countries.
2. It synthesizes a large (and growing) corpus of EGH literature, facilitating navigation to find deeper description of these themes in specific countries.

Additionally, countries can learn from and potentially adapt lessons from this study given the identified top drivers are often overarching issues that all nations must consider in their policy, strategy and programming decisions. For example, investments in data systems–despite varying contexts and challenges that countries experience, our study promotes the investment in rigorous data collection systems and its use for decision-making for achieving broader health goals. Details on how countries achieved these successes, as provided on the EGH platform and published studies, provide further guidance to countries on how to best design goals and activities for the driver. Certainly, the top drivers identified in this study are cross-cutting issues that would be helpful when countries are considering broader health and development agendas such as the Sustainable Development Goals or Universal Health Coverage. Whether its investments in data systems, coordinating key stakeholders, political commitment to the goals/plans, engaging and empowering women, or ensuring sustainable financing–by prioritizing these areas, EGH findings posit that the goals would be set up for success.

### Strengths and limitations

Global health research that employs a positive outlier lens can uncover valuable lessons that are less apparent through other approaches. For example, observational analysis of this sort can include complex driving factors that may have an effect over a long period of time, in comparison to experimental and quasi-experimental designs that often must be limited to shorter periods of observation and simpler causal factors, especially those that are more amenable to direct intervention. A focus on positive outliers specifically serves to efficiently narrow the scope of observational analysis to places that are known to have succeeded, thus narrowing research findings to effective interventions and strategies by extension.

However, positive outlier studies pose their own challenges. Identification of positive outliers can be complex and subject to measurement error since definitions and quantification of success may vary widely across topics and geographic units of study. Complicating the identification of outliers further, the EGH studies included here intentionally included countries with varied geographic and other characteristics, in some cases eschewing some outliers in the interest of regional representativeness. Furthermore, given the number of real-world factors that could influence outcomes, it may be more difficult to confidently identify causality. A positive outlier approach alone also can fail to include a counterfactual; it merely focuses on description of what *happened*, not what was *unique* about a successful location (compared to a less successful location). Future EGH studies may seek to include counterfactual (i.e., non-Exemplar) settings as a basis for comparison. Finally, a focus on positive outliers can give undue weight to successes while ignoring potential failures, even within positive outlier countries. Results need to be presented with full context so that readers can understand exactly which factors contributed and in what way.

Certainly, previous studies that have applied the positive deviance approach [2–6, 85, 86] have generally been able to identify key drivers for success in topics of study, while also uncovering bottlenecks or challenges, and making recommendations for policy and action–despite varying in their methodology and rigor. EGH tried enhancing this approach by applying a standardized, comprehensive methodological framework for all topics studied, undertaking several complementary qualitative and quantitative analyses to test sensitivity of findings, drawing on expert consultations throughout the research process, engaging policy and decision maker from the outset of the study design to results interpretation, and acknowledging the remaining challenges and counterfactuals.

The content analysis conducted here poses its own strengths and weaknesses as well. It can be an effective tool to provide new insights and improve a researcher’s understanding of a particular phenomenon [88] and can make it possible to convert words into content-related categories [46]. Yet, the method has been criticized for being simplistic and lacking detailed statistical analysis, for not being sufficiently qualitative in nature, and for being relatively subjective [46]. However, for this exploratory hypothesis-generating study, we feel the method was robust and sufficient, and we tried to mitigate the risk of subjectivity by having multiple content reviewers and validations with topic and study experts throughout the process.

A final limitation relates to the Exemplar studies used in the analysis. While the studies generally have a common mixed methods approach, the individual frameworks and methodologies used to derive study results vary and may not be exactly comparable to one another for this content analysis. Study periods also varied from a few years (during the COVID pandemic) to decades (stunting), though the major drivers were consistent between studies, nonetheless. The scope of this study also excluded deep examination into the studies that did *not* include key themes; for example, three studies did not report political commitment as a key theme. Future analysis may explore such divergence. However, the goal of this study was to highlight top drivers across the successes irrespective of the approaches used for research; it is our understanding that individual research partners would use the most rigorous, exhaustive, and evidence-informed frameworks, datasets, and methodologies to determine success factors for their topics and content analysis meta-analyzed for topline findings. In that regard, it is our understanding that findings from this content analysis are methodologically sound and are reflective of the overarching common themes among global health successes.

Our sensitivity analyses focused on the country (n = 19) rather than the country-study as the unit (n = 31), verifying the results identified in this work and strengthening the main messages we have reported.

### Future research

Further work will explore counterfactual Exemplar studies to understand “what doesn’t work” to drive national gains while exploring any subnational successes. This analysis of counterfactuals will also be used to contrast and compare cross-cutting challenges along with the cross-cutting success factors identified through this study. Further work will also explore instances where these cross-cutting themes did not emerge to understand how Exemplar countries achieved success without these factors to shed light on those studies where specific constructs were not studied or analyzed. Although we are not able to rank prioritize the set of cross-cutting drivers identified in this study, we will endeavor to explore the feasibility of such an assessment in future Exemplar multi-country analyses. Relatedly, countries and experts often are faced with the reality of identifying the relative costs and efficiencies of one intervention compared to another–while this analysis sheds some light on the key drivers, we were not able to link to costs or cost-effectiveness of these interventions. Future analyses will aim to explore this further.

## Conclusion

Resources for development are severely limited, and inadequate funding hinders the ability to fully implement every policy, strategy, and program. Within this universal constraint, however, some countries are able to spend more on key development priorities, some are able to maximize the impact of what they spend, and some are able to do both. We hope that by summarizing the common factors among the countries who have been able to maximize impact, findings from this study can aid funders, policymakers, and other stakeholders in understanding strategies to achieve optimal outcomes across health and human development.

## Supporting information

S1 File Acknowledgments. (DOCX)

S2 File Analysis of themes. (XLSX)

S3 File Standards for Reporting Qualitative Research (SRQR) checklist. (DOCX)
