# Supplementary material for: Drivers of success in global health outcomes: A content analysis of Exemplar studies
Source: PLOS Glob Public Health. 2024 May 9;4(5):e0003000. doi: 10.1371/journal.pgph.0003000 (PMC11081220; doi:10.1371/journal.pgph.0003000)
Supplement: S1 File — (DOCX) [file pgph.0003000.s001.docx]

Drivers of success in global health outcomes: a content analysis of Exemplar studies

S1 File – Acknowledgements

Membership of the Exemplars in Global Health Partner Network includes the following individuals: Maiga Abdullaye, Kriti Adhikari, Mengesha Admassu, Kaosar Afsana, Justice Moses Aheto, Nadia Akseer, Mohammad Ariful Alam, Sarasi Amarasinghe, Alemayehu Amberbit, Agbessi Amouzou, Anushka Ataullahjan, Sasmrita Bastola, Robert A Bednarczyk, Sara Bennett, Caroline Beyer, Rachid Bezad, Basudev Bhattarai, Zulfiqar A Bhutta, Agnes Binagwaho, Robert Black, Andrea Blanchard, James Blanchard, Hannah Blencowe, Ties Boerma, Vera Joanna Bornstein, Niranjan Bose, Samanpreet Brar, Himanshu Bushan, Oona MR Campbell, Marco H Carcamo, Carla López Castañeda, Francisco Castillo-Zunino, Francesca Cavallaro, Kimberly Charbonneau, Elisa Juarez Chavaz, Paul Li Jen Cheh, Nan Chen, Mushtaque Chowdhury, Melanie Coates, Erica Confreda, Kaitlin Conway, Bernice Dahn, Shanti Dalpatadu, Raja Ram Dhungana, Mareme Diallo, Ibrahima Diouf, Sameer M Dixit, Kateri Donahoe, Bonheur Dounebaine, Laura Drown, Duah Dwomoh, Shams El Arifeen, Anna S Ellis, Zalina Enikeeva, Karl Everett, Omar Faruk, Cheikh Faye, Julius Fobil, Sachini Fonseka, Matthew C Freeman, Miriam Frisch, Jacopo Gabani, Patricia J Garcia, Zaira Gasanova, Seifu Gebreyesus, Azrah Ghaffoor, Argie Gingoyon, Camila Giugliani, K Madan Gopal, Moytrayee Guha, Claire Gwayi-Chore, Luidina Hailu, Navodi Mekhala Hakmanage, Manzoor Ahmed Hanifi, Piya Hanvoravongchai, Elizabeth Hazel, Nehmat Helou, Kyra Hester, Lisa R Hirschhorn, Aniqa Tasnim Hossain, Fauzia Akhter Huda, Luis Huicho, Zahra Hussain, Mariia Iamshchikova, Issah Ibrahim, Gloria Ikilezi, Shajy Isac, Muhammad Islam, Rachel Jardine, Safia Jiwani, Tanya Jones, Heather Jue-Wong, Aminata Ka, Steven Ndugwa Kabwama, Malick Kante, Emily Keats, Pinar Keskinocak, Sirithorn Khositchaiwat Khositchaiwat, William Kilembe, Meredith Kimball, Jessica King, Dilbara Kirbasheva, Suzanne Narayan Kiwanuka, Susan Kizito, Adam Koon, Nalin Kumara, Youssoufa Lamou, Isabelle Lange, Wasin Laohavinij, Karin Lapping, Carlile Lavor, Stephen S Lim, Anne Liu, Hassan Rushekh Mahmood, Abdoulaye Maiga, Anustha Mainali, Melisa Martinez-Alvarez, Mahesh Maskey, Kedest Mathewos, Andrea Meeson, Dessalegn Melesse, Katie Micek, Roman Mogilevskii, Afrah Mohammedsanni, Fred Monje, Claudio A Mora-García, Lucia Mullen, Shegaw Mulu, Melinda Munos, Kyle Muther, Alice Namale, Noel Namuhani, Dima Nazzal, Rawlance Ndejjo, Dhanusha Nirmani, Jovial Thomas Ntawukuriryayo, Jennifer Nuzzo, Will Oswald, Raj Panjabi, Ian Paulino, Andy A Pearson, Loveday Penn-Kekana, Sanjay Perera, Udita Persaud, David E Phillips, Aungsumalee Pholpark, Catherine Pitt, Nikita Pradhan, Andrea M Prado, Matt Price, Ahmed Ehsanur Rahman, Rajesh M Rajbhandari, Usha Ram, BM Ramesh, Ravindra P Rannan-Eliya, Aviva Rappaport, Magdalena Rathe, Laura Rathe, Natchaya Ritthisirikul, Zoe Sakas, Mohamadou Sall, Sarath Samarage, Moussa Sarr, Felix Sayinzoga, Kerry E Scott, Anjana Senadeera, Ibrahima Sene, Sudha Sharma, Sonya Shekhar, Bilal Shikur, Neha Singh, Ishwari Sivagnanam, Paola Solda, Brendan Sorichetti, Raj Kumar Subedi, Thanathip Suenghataiphorn, Ashenif Tadele, Yvonne Tam, Arielle Cohen Tanugi-Carresse, Hana Tasic, Alula M Teklu, Dip Narayan Thakur, Andrew K Tusubira, Kelechi Udoh, Tyler Vaivada, Amelia VanderZanden, Elisa Vidal, Yohannes Wado, Neff Walker, Shelley Walton, Rhoda K Wanyenze, Jannah Wigle, Nilmini Wijemunige, Anna Larson Williams, Niroshani Wisidagama, Kerry Wong, and Isaac Yeboah.
