# Supplementary material for: Drivers of success in global health outcomes: A content analysis of Exemplar studies
Source: PLOS Glob Public Health. 2024 May 9;4(5):e0003000. doi: 10.1371/journal.pgph.0003000 (PMC11081220; doi:10.1371/journal.pgph.0003000)

**Standards for Reporting Qualitative Research (SRQR)**

| **No.** | **Topic** | **Addressed** | **Location in Manuscript** | **Notes** |
| --- | --- | --- | --- | --- |
| S1 | Title |  | Pg.1 |  |
| S2 | Abstract |  | Pg. 1-2 |  |
| S3 | Problem formulation |  | Pg. 2-4 | “One effective model for generating such evidence is known as a positive deviance, or positive outliers approach, whereby places, programs, or projects that have had exceptional success relative to their economic status are identified and studied for transferrable lessons [1].”  “This approach has been applied in global health in the past, with varying degrees of rigor [2–6].”  “To date, the EGH program has studied, or is currently studying, 14 major global health topics…”  “…to date, no systematic effort has been taken to summarize findings across [EGH] topics.” |
| S4 | Purpose or research question |  | Pg. 4 | Final paragraph before Materials and Methods |
| S5 | Qualitative approach and research paradigm |  | Pg. 5  Pg. 8  Pg. 8 | First sentence  First paragraph under Analysis – includes rationale for content analysis: “Content analysis is a flexible approach that is well-suited for analyzing data on multifaceted phenomena…”  Paragraph under Conceptual framework |
| S6 | Researcher characteristics and reflexivity |  | Pg. 9  Pg. 10 | “The analysis process was led by NA with continuous consultation with internal EGH research teams and additional consultation with research teams responsible for the original studies.”  Paragraph under validation |
| S7 | Context |  | Pg. 6  Pg. 6-7 | “We conducted a desk review of all completed EGH country case study reports, online platform narratives, and peer-reviewed publications for the 31 completed studies.”  Table 2: study countries by topic |
| S8 | Sampling strategy |  | Pg. 5 | “We used purposive sampling, considering all completed EGH studies (N= 31) from June 2020 to May 2023 for inclusion in this analysis. Studies were excluded if validation of final results was ongoing or if research partners requested to independently publish findings prior to this study.” |
| S9 | Ethical issues pertaining to human subjects |  |  | Not applicable, as study was based on secondary analysis of published data. |
| S10 | Data collection methods |  | Pg. 5  Pg. 6 | “…considering all completed EGH studies (N=31) from June 2020 to May 2023…”  Paragraph under Data sources |
| S11 | Data collection instruments and technologies |  | Pg. 9 | “Data coding was performed manually, and descriptive analyses were executed in Excel 2023 and Stata version 15.” |
| S12 | Units of study |  | Pg. 6 | “This included a total of 70 documents across the six topics.” |
| S13 | Data processing |  | Pg. 8 | “We initially utilized an inductive approach (i.e., open coding), followed by deductive coding that was guided by the conceptual framework [47]. After coding, data were then categorized and mapped in larger categories and grouped into emergent themes to identify top drivers of the successes achieved in Exemplar studies (i.e., factors that were critical or catalytic in achieving the intended outcome) [47].” |
| S14 | Data analysis |  | Pg. 9 | First paragraph |
| S15 | Techniques to enhance trustworthiness |  | Pg. 9  Pg. 9  Pg. 10 | “The analysis process was led by NA with continuous consultation with internal EGH research teams and additional consultation with research teams responsible for the original studies. Any discrepancies were discussed and resolved by cross-referencing study documents and consultations with research partners, where needed.”  “Sensitivity analyses were also conducted…”  Paragraph under Validation |
| S16 | Synthesis and interpretation |  | Figure 3  Pg. 11-18 | Key Themes and Sub-themes from EGH studies  Summary of major themes (what Exemplars did) and key sub-themes (how they achieved success in each area) |
| S17 | Links to empirical data |  | Pg. 11-18 | Each theme / sub-theme includes 1-4 country-specific examples |
| S18 | Integration with prior work, implications, transferability, and contribution(s) to the field |  | Pg. 19-21 | From “These findings generally align with previous efforts aimed at identifying drivers of global health improvements…” to Implications of findings section |
| S19 | Limitations |  | Pg 21-23 | Under Strengths and limitations |
| S20 | Conflicts of interest |  |  | N/A  Separate conflict of interest forms will be filled out for each study author |
| S21 | Funding |  |  | Funding statement is included in the final publication |

Reference:

O’Brien BC, Harris IB, Beckman TJ, et al. Standards for Reporting Qualitative Research: A Synthesis of Recommendations. Academic Medicine 89(9):1245-51, 2014.


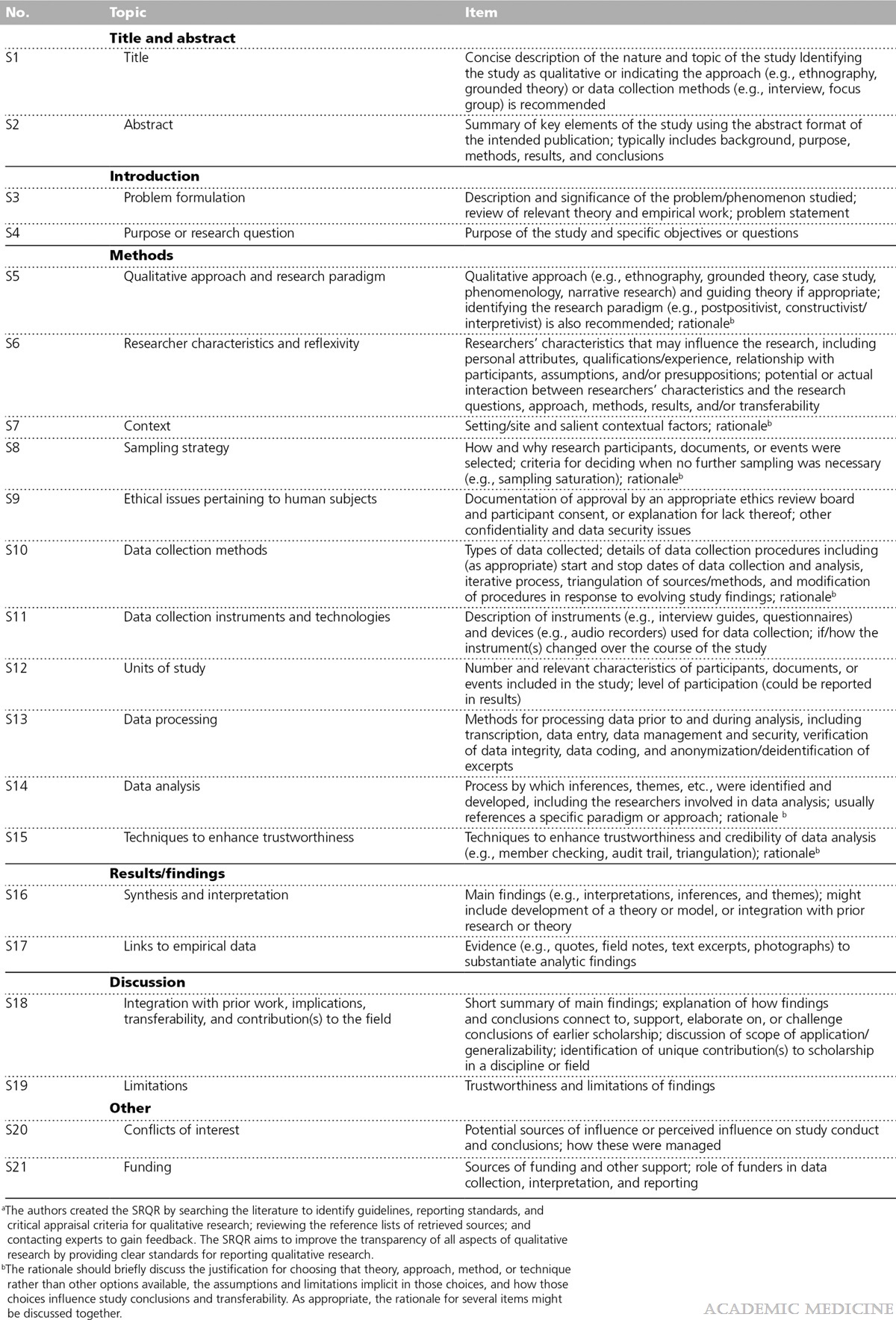

Supplement: S3 File — (DOCX) [file pgph.0003000.s003.docx]
